# Supplementary material for: Pressure sensing mat as an objective and sensitive tool for the evaluation of lameness in rabbits
Source: PLoS One. 2023 Jul 7;18(7):e0286918. doi: 10.1371/journal.pone.0286918 (PMC10328299; doi:10.1371/journal.pone.0286918)
Supplement: S1 Table — (PDF) [file pone.0286918.s001.pdf]

Single values of $ratio_{pressure}$

measurement day	animal number											
	1	***2***	***3***	***4***	***5***	***6***	***7***	***8***	***9***	***10***	***11***	***12***
1st measurement day	-0.20<br>0.08<br>0.05<br>0.03	-0.05<br>0.09<br>0.17	0.04<br>-0.13<br>0.17<br>-0.13<br>-0.35	0.15<br>0.10<br>0.29<br>-0.05<br>0.06	-0.04<br>0.15<br>-0.31<br>0.10	0<br>-0.02<br>0.01<br>-0.13<br>0.04	-0.12<br>0.02<br>-0.24<br>0.06<br>-0.19	0.04<br>-0.04<br>0.08<br>-0.13	0<br>-0.20<br>-0.04<br>0.21<br>-0.04	-0.06<br>-0.08<br>-0.13<br>0.07<br>0.10	-0.33<br>0.06<br>-0.12<br>-0.08<br>0.01	0.14<br>0.02<br>-0.10<br>-0.10<br>-0.05
week 1	-0.52<br>-0.54	-0.18<br>-0.37<br>-0.55	-0.74<br>-0.61<br>-1.00	-0.74<br>-0.76<br>-1.00	-0.48<br>-0.39<br>-0.44<br>-0.50	-0.68<br>-0.56<br>-0.65<br>-0.55	-0.65<br>-0.24<br>-0.51<br>0.15	-1.00<br>-0.81<br>-1.00	-1.00<br>-0.32<br>-0.11<br>-0.59	-0.81<br>-0.71<br>-0.83	-0.68<br>-0.22<br>-0.05<br>-0.03	-0.68<br>-0.51<br>-0.67
week 12	-0.24<br>0.04<br>-0.34	-0.43<br>-0.29<br>-0.32<br>-0.08<br>-0.28<br>-0.30	-0.29<br>-0.05<br>-0.18<br>0.03	-0.27<br>-0.13<br>-0.46<br>-0.28<br>-0.28	-0.20<br>-0.29	-0.15<br>-0.21<br>-0.16<br>-0.35<br>-0.13	-0.14<br>-0.26<br>-0.18<br>-0.23	-0.12<br>-0.15<br>-0.27<br>0.10	0.20<br>-0.45	0.04<br>-0.06<br>-0.03	-0.33<br>-0.21	0.06<br>-0.10<br>-0.07<br>-0.13

Single values of $ratio_{force}$

measurement day	animal number											
	1	***2***	***3***	***4***	***5***	***6***	***7***	***8***	***9***	***10***	***11***	***12***
1st measurement day	-0.24<br>0.17<br>-0.13<br>0.15	-0.07<br>0.11<br>0.16	0.23<br>0.08<br>0.20<br>-0.16<br>-0.32	0.16<br>-0.09<br>0.15<br>-0.03<br>0.08	0.22<br>0.04<br>-0.10<br>0.23	0.07<br>-0.07<br>-0.06<br>0.02<br>-0.09	-0.06<br>-0.03<br>-0.34<br>0.08<br>0.06	-0.10<br>0.10<br>0.01<br>-0.16	-0.11<br>-0.06<br>0.08<br>0.05<br>0.04	0.02<br>-0.09<br>-0.15<br>0.02<br>0.05	-0.21<br>0.18<br>0.13<br>-0.30<br>-0.26	0.21<br>-0.01<br>-0.07<br>-0.05<br>0.08
week 1	-0.84<br>-0.94	-0.85<br>-0.68<br>-0.76	-0.93<br>-0.77<br>-0.89	-0.95<br>-0.97<br>-1.00	-0.94<br>-0.96<br>-1.00<br>-0.90	-0.81<br>-0.86<br>-0.82<br>-0.90	-0.85<br>-0.87<br>-0.75<br>-0.81	-0.50<br>-0.70<br>-0.16	-1.00<br>-0.98<br>-1.00<br>-1.00	-0.59<br>-0.41<br>-0.63	-0.93<br>-0.92<br>-0.94<br>-0.96	-0.29<br>-0.33<br>-0.32
week 12	-0.16<br>-0.20<br>-0.28	-0.45<br>-0.57<br>-0.33<br>-0.42<br>-0.45<br>-0.61	-0.55<br>-0.04<br>-0.48<br>-0.09	-0.54<br>-0.33<br>-0.59<br>-0.63<br>-0.58	-0.62<br>-0.50	-0.33<br>-0.49<br>-0.24<br>-0.50<br>-0.27	-0.28<br>-0.13<br>-0.24<br>-0.20	-0.07<br>-0.14<br>-0.20<br>0.06	-0.28<br>-0.60	-0.05<br>-0.19<br>-0.16	-0.55<br>-0.36	-0.18<br>-0.16<br>-0.13<br>-0.33

Mean values of $ratio_{pressure}$

measurement day	animal number											
	1	***2***	***3***	***4***	***5***	***6***	***7***	***8***	***9***	***10***	***11***	***12***
1st measurement day	-0.01	0.07	-0.08	0.11	-0.02	-0.02	-0.09	-0.01	-0.01	-0.02	-0.09	-0.02
2nd measurement day	-0.15	-0.07	0.02	-0.02	-0.05	-0.03	-0.12	0.05	-0.09	0.02	0.04	-0.04
3rd measurement day	-0.03	0.05	0.04	0.02	-0.05	0.04	-0.09	0.11	-0.03	0.03	-0.08	-0.04
preoperative	-0.06	0.02	0	0.04	-0.04	0	-0.10	0.05	-0.04	0.01	-0.04	-0.03
week 1	-0.53	-0.37	-0.62	-0.78	-0.74	-0.50	-0.60	-0.20	-0.95	-0.34	-0.76	-0.10
week 12	-0.18	-0.28	-0.12	-0.29	-0.24	-0.20	-0.20	-0.11	-0.13	-0.01	-0.27	-0.06

Mean values of $ratio_{force}$

measurement day	animal number											
	1	***2***	***3***	***4***	***5***	***6***	***7***	***8***	***9***	***10***	***11***	***12***
1st measurement day	-0.01	0.07	0.01	0.06	0.10	-0.03	-0.06	-0.03	0.00	-0.03	-0.09	0.03
2nd measurement day	-0.20	-0.09	0.02	0.01	-0.14	-0.12	-0.10	0.19	-0.08	0.06	-0.01	-0.14
3rd measurement day	-0.09	-0.03	0.00	-0.01	-0.22	0.02	0.02	-0.02	0.08	0.00	-0.08	-0.11
preoperative	-0.10	-0.02	0.01	0.02	-0.08	-0.06	-0.05	0.05	0	0.01	-0.06	-0.07
week 1	-0.89	-0.76	-0.86	-0.97	-0.95	-0.85	-0.82	-0.45	-1.00	-0.55	-0.94	-0.31
week 12	-0.21	-0.47	-0.29	-0.54	-0.56	-0.36	-0.21	-0.09	-0.44	-0.13	-0.45	-0.20
