# Supplementary material for: Pressure sensing mat as an objective and sensitive tool for the evaluation of lameness in rabbits
Source: PLoS One. 2023 Jul 7;18(7):e0286918. doi: 10.1371/journal.pone.0286918 (PMC10328299; doi:10.1371/journal.pone.0286918)
Supplement: Attachment — Submitted filename: Response to Reviewers.docx [file pone.0286918.s002.docx]

First, we thank the reviewers for their valuable comments.

In the following, we responded to each comment point by point. Changes to the paper are highlighted in yellow.

**Reviewer 1**

at line 115 is not clear if also the knee affected by the surgery was mono or bilateral for each rabbit

We changed the position of the word “right” to clarify this.

*“Based on another OA-study rabbits got a surgical intervention at the right knee joint, including mechanical destabilization of the joint by transection of the anterior cruciate ligament (ACLT) and incision of the medial meniscus aiming to achieve the development of post-traumatic osteoarthritis (26).”*

at line 133 and 141, the authors should indicate the level of experience of veterinarians

The principal veterinarian of the study works in the field of orthopaedic animal research with rabbits (and other animal models) for over 15 years and is highly experienced consequently. The other veterinarians involved in the scoring of the animals all also joined the pilot study and therewith are also experienced personnel. To minimize variance of score values, the scoring was performed together when the study started to train all involved persons to the same principles. In the subsequent study, the principal veterinarian spot-checked the given values.

Due to comments of reviewer 2, this paragraph was shortened and slightly restructured. “Experienced” was added and a sentence about how to ensure maximum inter-observer agreement was also added.

The paragraph:

*“The pain was evaluated by visual gait analysis, which was performed on each rabbit with a scoring scheme consisting of a point system (Table 1), we also used in a previous study [26]. It was used by veterinarians of the working group in familiar surroundings in the stable room under constant conditions. Visual assessment of the lameness was complemented by an orthopaedic examination, which includes the passive range of mobility and pain expression shown by paw withdraw or vocalisation during careful touching and passive motion of both knee joints in comparison (Table 1). Clinical scoring was performed daily for the first two weeks and subsequently three times a week. On pressure sensing mat measurement days, the orthopaedic examination was performed in the setup, to ensure the animals were not treated more than necessary. Due to daily work routines, four different veterinarians performed the examinations in turns, all of whom were trained according to the same principles.”*

was changed to:

*“The pain was evaluated by visual gait analysis, which was performed on each rabbit with a scoring scheme consisting of a point system (Table 1), as previously described (26) in familiar surroundings in the stable room under constant conditions. ~~Visual assessment of the lameness was complemented by an orthopaedic examination, which includes the passive range of mobility and pain expression shown by paw withdraw or vocalisation during careful touching and passive motion of both knee joints in comparison (Table 1).~~ Clinical scoring was performed daily for the first two weeks and subsequently three times a week. On pressure sensing mat measurement days, the clinincal scoring was performed in the setup, to ensure the animals were not treated more than necessary. Due to daily work routines, four experienced veterinarians performed the examinations in turns, all of whom were trained according to the same principles. To maximize inter-observer agreement, scoring at the start of the study was performed together with subsequent spot-check inspections by the principle veterinarian.”*

at line 172, the authors should specify if they had set a time limit within which the five trials could be obtained.

No exact time limit was set, but the measurements were performed calmly and as quickly as possible. The measurement was abandoned ahead of reaching five trials if the rabbit could not or only with considerable effort be motivated to hop over the mat and/or obvious evidence of stress was noticeable.

We added a respective sentence to the paragraph:

“*Aim was to obtain five successful measurements per animal per day. The evaluation was abandoned ahead of reaching five trials if the rabbit could not or only with considerable effort be motivated to hop over the mat and/or obvious evidence of stress was noticeable. Measurements fulfilling the following criteria were considered successful:”*

At line 143: table 1 orthopedic score

- grade 2 and 3 must be better defined, it is not clear what the difference between the two scores is because it is not clear what "low-grade algesia" and "middle-grade algesia" mean
-10 which means expression of pain without palpation? What parameters or clinical signs were considered?

Due to your comment and the comment of reviewer 2 about the problems with the orthopaedic score and since this score had no further meaning to this paper, it was deleted from the paper.

Given the paucity of literature, the discussion fails to justify some points fully; in particular, I believe that the stimulus given to walking animals that tended not to walk may, in some way, affect their gait.

Thank you for your valuable comment. Prompted walking is often part of gait analysis in animals, as for example in automated catwalk systems using treadmills where the animals have to walk due to the moving belt underneath. We agree that “induced” walking may lead to a somehow altered gait pattern compared to spontaneous walking. Especially the fact, that rabbits are flight animals may influence their gait when induced externally. When adrenalin rushes in due to escape reflex, sensation of pain might be suppressed. In our experimental set up, escape reflexes were immaterial. Many cases of rabbits that needed a stimulus to walk were due to the fact, that the animals were very well accustomed to the veterinarians and simply saw no reason to run away from them. Also, it occurred repeatedly that the hind legs of the rabbits did not hit the pressure sensor mat because they hopped over it and consequently the run had to be repeated. Other reasons for “not successful runs” were “only one hind paw hit the mat”, “rabbit stamped on the mat”, “rabbit paused or stopped on the mat” with subsequent need for repetition. After a couple of repetitions, their will to run voluntarily just faded and they had to be shooed. Also – depending on the character of the single animal – stress symptoms increased with increasing amount of repetitions.

However, after prompting, the veterinarian did not affect the run itself. If the gait pattern over the mat was not continuously (e.g. stopping, or stamping with one hind paw or similar events), again the run was not included.

The subjective gait pattern which was examined through the lameness scoring in the stable on the same day was also taken into account. No obvious differences were noticed for all rabbits in the way they ran between stable and the pressure sensor mat set up, also after prompted walking/running.

Beside the studies referenced within our paper, there are numerous papers which report on different gait analysis systems using voluntary movement as in open fields or catwalk systems, or forced movement as in automated systems using treadmills (e.g. DigiGait™, True Treadmill), for example

1. Lambert CS, Philpot RM, Engberg ME, Johns BE, Kim SH, Wecker L (2014): Gait Analysis and the Cumulative Gait Index (CGI): Translational Tools to Assess Impairments Exhibited by Rats with Olivocerebellar Ataxia. Behav Brain Res. 2014 November 1; 0: 334–343. doi:10.1016/j.bbr.2014.08.004.
2. Neumann M, Wang Y, Kim S, Hong SM, Bilgen M, Liu J (2009): Assessing gait impairment following experimental traumatic brain injury in mice. J Neurosci Methods. January 15; 176(1): 34–44. doi:10.1016/j.jneumeth.2008.08.026.
3. Petkova SP, Adhikari A, Berg EL, Fenton TA, Duis J, Silverman JL (2022): Gait as a quantitative translational outcome measure in Angelmansyndrome. Autism Research.2022;15:821–833.
4. Deuis J, Dvorakova LS, Vetter I (2017): Methods Used to Evaluate Pain Behaviors in Rodents. Front. Mol. Neurosci. 10:284. doi: 10.3389/fnmol.2017.00284.

Petkova et al. are one of the few references who used different gait analysis techniques for the same study. However, they only report on the different parameters which resulted from the regarding technique but did not comment on advantages and disadvantages of one method over the other.

The review by Deuis et al. (2017) succeeds in presenting different set ups for the evaluation of pain including a paragraph on weight bearing and gait analysis. They also assess the different advantages and disadvantages. Although they point out the value of unrestrained pain evaluation in animals, they do not comment on the impact of forced motion.

Although we also believe, that unrestrained gait analysis would be superior to our presented set up, realization in a rabbit model as used here is difficult. While treadmills are used for rabbits in studies including cardiovascular subjects, automated treadmills with cameras for gait analysis as used for mice and rats are not established yet for the rabbits. The group by Juncosa et al. (reference 23 in our paper) used a treadmill, which is commonly used in humans with further adaption to make it “animal safe” using a custom-designed, clear, polycarbonate enclosure. The rabbits only performed three hops inside this enclosure, of which the middle one was chosen to evaluate. Although having a constant treadmill speed, the rabbits themselves did not move at a constant speed. Furthermore, no ground reaction forces could be measured since the animals did not like to hop on the Tekscan measuring mat.

Taken all these information into account, we added the following part to the discussion:

*“However, as described in the methods, rabbits might need to be motivated by the experimentator to hop which could potentially influence the gait pattern. Beside the already referenced literature, there are numerous papers, which report on different gait analysis systems using either voluntary movement as in open fields or catwalk systems, or forced movement as in automated systems using treadmills (e.g. Lambert, Neumann, Petkova, Deuis). Petkova et al. are one of the few references who used different gait analysis techniques for the same study. However, they only report on the different parameters which resulted from the regarding technique but did not comment on advantages and disadvantages of one method over the other.*

*The review by Deuis et al. (2017) succeeds in presenting different set ups for the evaluation of pain including a paragraph on weight bearing and gait analysis. They also assess the different advantages and disadvantages. Although they point out the value of unrestrained pain evaluation in animals, they do not comment on the impact of forced motion.*

*Although we also believe, that unrestrained gait analysis would be superior to the here presented set up, realization in a rabbit model as used here is difficult. While treadmills are used for rabbits in studies including cardiovascular subjects, automated treadmills with cameras for gait analysis as used for mice and rats are not established yet for the rabbits. The group by Juncosa et al. (reference 23 in our paper) used a treadmill, which is commonly used in humans with further adaption to make it “animal safe” using a custom-designed, clear, polycarbonate enclosure. The rabbits only performed three hops inside this enclosure, of which the middle one was chosen to evaluate. Although having a constant treadmill speed, the rabbits themselves did not move at a constant speed. Furthermore, no ground reaction forces could be measured since the animals did not like to hop on the Tekscan measuring mat (23). measuring mat. In our study, after prompting, the veterinarian did not affect the run itself. If the gait pattern over the mat was not continuously (e.g. stopping, or stamping with one hind paw or similar events), again the run was not included. Therewith, we assess the influence of this moving stimulus as negligible.”*

**Reviewer 2**

The syntax makes some sentences very difficult to read and the help of a professional editorial service would benefit the entire manuscript.

Thank you for your advice, the manuscript was checked again thoroughly and changes were done throughout the text, especially in the discussion. To preserve readability and detection of the changes for the other comments, language changes are not highlighted.

The caption for your figures are imbedded in the manuscript and not repeated below the figures in pages 29-34, which makes it harder to connect.

We are sorry, that it is hard to connect and agree with you. However, these are the submission guidelines of PLOS ONE and we cannot not change the position of the captions in the manuscript.

Line 26 Specify Unilateral

Although line 26 did not contain “unilateral” we believe you request a specification of the implantation site. We added “of the right knee”.

*“They got an artificial anterior cruciate ligament transection of the right knee in connection with…”*

Line 35-37: rephrase, the sentence does not read well.

The sentence was rephrased as follows:

*“In week one, the visual score resulted in a mild to moderate lameness and in week twelve with*

*exception of one, rabbits were evaluated as lame free. Contrary, the sensor mat showed low*

*grade lameness in almost all rabbits in week twelve and in week one, lameness appeared to*

*be more pronounced.”*

was changed to:

*“Following the visual score, lameness in week one was mild to moderate. In week twelve, rabbits were evaluated as lame free bar one. Contrary, following the values of the sensor mat, lameness in week one appeared to be more pronounced and almost all rabbits still showed low-grade lameness in week twelve.”*

Line 38: delete "it can", conclusions following appropriate experiment and statistical analysis are definitive.

“It can” was changed to “Consequently”.

Line 62-63: Proposal: Video recording using cameras have been described and used to overcome the subjectivity and poor interobserver consistency of visual lameness scoring systems.

The proposal has been included as follows:

*“Video recording using cameras have been described and used to overcome the subjectivity and potentially poor interobserver consistency of visual lameness scoring systems.”*

Line 63 delete "e.g.".

“e.g.” was deleted.

Line 64: reflective markers assigned to bone and joints: kinematics.

Thank you for this proposal. We agree, that generally, “reflective markers assigned to bone and joints” quasi equals to the term “kinematics”. However, since this sentence refers to a specific paper, which does not use the term “kinematics” at all, we did not use it in this context either.

Line 87: Pressure mat are extensively used and their reliability is known in many species. Please cite few studies here that used this device as a gait analysis tool (e.g.: Steiner, R., Dhar, M., Stephenson, S.M., Newby, S., Bow, A., Pedersen, A. and Anderson, D.E., 2019. Biometric Data Comparison Between Lewis and Sprague Dawley Rats. Frontiers in Veterinary Science, 6, p.469.
/ Rifkin RE, Grzeskowiak RM, Mulon PY, Adair HS, Biris AS, Dhar M, Anderson DE. Use of a pressure-sensing walkway system for biometric assessment of gait characteristics in goats. PloS one. 2019 Oct 16;14(10):e0223771.)

Thank you for the suggested paper. We included them in the paper and slightly adapted the wording of the following sentence as follows:

*“Pressure mats are extensively used and their reliability is known in many species (22,27–29). Since sensors that fit to the size and weight of rabbits are commercially available, their application in rabbit models seems promising.”*

Line 91-95: Structure this sentences in goals/aims of the study and hypothesis related to each aim.

The sentences:

*“This study is focused on implementation this objective lameness assessment into routine controls. The main hypothesis that lameness assessment with a pressure sensing mat is more sensitive than visual scoring in rabbit lameness analysis should be verified. Finally, the study aimed to establish the method as a standardized in vivo method of gait analysis in rabbits for various types of orthopaedic research and others.”*

has been restructured to:

*“The aim was to establish the method as a standardized in vivo method of gait analysis in rabbits for various types of orthopaedic research and others. Therefore, the main hypothesis that lameness assessment with a pressure sensing mat is more sensitive than visual scoring in rabbit lameness analysis should be verified and aspects of implementation in routine control should be identified.”*

Line 109-110: rephrase

The sentence was deleted.

The bodyweight has been introduced earlier in the paper at line 104:

Sentence before:

*“Growing up to an age of twenty-four weeks, they were housed in their constant group of 12 animals in constant conditions (RT 18°C +/- 3°C, humidity 55% +/- 10%) in ground housing on coarse shavings.”*

Sentence now:

*“Growing up to an age of twenty-four weeks with an average bodyweight of 4.08 kg ± 0.45, they were housed in their constant group of 12 animals in constant conditions (RT 18°C +/- 3°C, humidity 55% +/- 10%) in ground housing on coarse shavings.”*

Line 117: description the manipulation of the meniscus: luxation, tearing, folding, detachment….

We replaced “*manipulation of the medial meniscus*” to “*incision in the medial meniscus*”.

Line 119-122: move this paragraph prior to the brief surgical description (line 115-118) to follow the chronological order of your experiment.

Thank you for this suggestion. The paragraph was brought forward by one paragraph, now starting at line 115.

Line 129: did you measure the inter-observer agreement?

Technically, the inter-observer agreement was not measured. Not all veterianarians scored at the same day. However, all were already involved in the scoring of the animals of the pilot study. The training at the beginning of the study was done in such a way that variance of score values was minimized. The principal veterinarian of the study (who works in the field of orthopaedic animal research with rabbits and other animal models for over 15 years) scored the animals together with the others when the study started to assure that everybody involved is trained to the same principles. In the subsequent study, the principal veterinarian spot-checked the given values.

In response to a comment of reviewer 1, we added a part about the inter-observer agreement at the end of this paragraph:

“*To maximize inter-observer agreement, scoring at the start of the study was performed together with subsequent spot-check inspections by the principle veterinarian.”*

Line 131: modify "we just used in a previous study" to "as previously described [26]."

We changed the sentence accordingly (highlighted in yellow).

Line 133: Passive range of motion

Due to deleting the orthopaedic score (see your last comment), the respective term is also deleted.

Line 139-140: What do you mean by "not treated more than necessary"? Same comment for the inter-observer agreement?

We apologize for not using clear wording.

To score the rabbits (apart from visual lameness score), they had to be catched out of the group, which is also necessary for the transportation to the neighboring room where the experimental set up for the pressure sensor mat was situated. We just wanted to point out that on days of sensor mat measurements the scoring was done in the other room instead of the stable.

Since this fact is not really of relevance to the paper, we deleted the respective sentence.

The paragraph now is as follows:

*“Clinical scoring was performed daily for the first two weeks and subsequently three times a week.* *~~On pressure sensing mat measurement days, the clinincal scoring was performed in the setup, to ensure the animals were not treated more than necessary.~~ Due to daily work routines, four different experienced veterinarians performed the examinations in turns, all of whom were trained according to the same principles. To maximize inter-observer agreement, scoring at the start of the study was performed together with subsequent spot-check inspections by the principle veterinarian.”*

Line 152: collocation?

The word was changed to “*arrangement*”.

Line 155: the larger areas had specified dimensions with known surface areas. Be more specific.

Due to cleaning of the room/environment, the set up dimensions were not totally static. Especially the front boundary had to be de-installed and re-installed occasionally. Therefore, we stated a range for the larger area.

Line 196: do you mean length or duration of contact?

We meant the duration of contact of the hind paw. The term has been replaced.

*“The integral of force over time during contact by the associated hind paw. Rationale: The rabbits might offload the affected paw by reducing the duration of contact without increasing the force.”*

Line 203: Comment on the standard deviation is a result and should be presented as a result and not in the M&M section.

After considering your comment and re-reading the paper we decided to delete the paragraph about the two other possible parameters (line 198 – 204) since they do not add substantially to the paper.

Line 206: it appears to have an extra parenthesis in your formula.

Thank you for pointing this out. We deleted the extra parenthesis.

Line 215: the lameness scores are categorical data and cannot be presented as mean +/- SD, please explain how you handled those results.

Thank you again for pointing this out and we totally agree with your comment. We have only presented metric data as mean and SD. The respective sentence has been changed as follows:

“*Mean values and standard deviation were determined for metric data.”*

Line 214-224: You choose to use a Wilcoxon test to compare the data of each rabbit, why did you chose to not use an ANOVA for the comparison of the data for each time points (pre-op, week 1 and week 12)?

The suggested approach by analyzing the results with ANOVA and treating the time points as an additional variable would indeed be feasible. However, especially considering the limited sample size, we chose the straightforward and non-parametric analysis of three separate Wilcoxon tests. If the reviewer insists, we could add a non-parametric Kruskal-Wallis test.

Line 291-292: while pressure mat is certainly a more objective tool to quantify the lameness/gait of animals when compare to visual assessment, the data you present do not indicate if the leg favoring indicated by the range of both ratio pressure and ratio force was consistent for every rabbit, or if it varies per passage for each individual. If the leg favoring was consistent for one individual, why was this rabbit not excluded from the study?
One criticism that is not addressed in your discussion is the possible inconstancy for the visual lameness score evaluators.

Figures 3a and 3c present the data of each single rabbit as boxplot for the first measurement day for ratio pressure and ratio force. Although the median values for one animal were either below or above zero thus indicating an overall favoring of one limb, the position of the boxes and whiskers demonstrate that they indeed alternately favor the limbs for the different runs that were basis for the calculation. That is also the reason why we recommend achieving a minimum of five successful runs and why we defined a physiological range instead of using zero.

Regarding the possible inconstancy for the visual lameness score evaluators we already described our effort to minimize the variance in our answer to your comment for line 129 (see above).

Beside these actions we agree that a hundred percent consistence between the different evaluators for the visual scoring cannot be guaranteed, which is – apart from the lower sensitivity – the reason to use other techniques as sensor mats.

We included a part to the discussion as follows:

*“A limitation of this study is that different veterinarians scored the animals on the different days thus potentially leading to inconsistent score values. Validity would be considerably enhanced if all experimentators would have scored at each day followed by subsequent calculation of the inter-observer agreement. Due to technical reasons, this was not possible. However, inter-observer agreement was maximized by I. including only veterinarians with experience in rabbit lameness evaluation, II. thorough training under supervision of the principal veterinarian at the start of the study and III. spot-check inspection of consensus by the principal veterinarian during the course of the study.”*

Line 393-394: Agree, however some refinement in your article are necessary.

We hope we have satisfactorily refined the article according to the aspects specified in your comments and our answers.

Table1: Why did you give a score of 10 instead of the following number (4 and 6) for the highest score? I would suggest to modify it understanding this is categorical data and not continuous data.

Within our application for the animal experiments we have to estimate the expected animal burden during the course of the experiment including a score system for all aspects (clinical signs of the wound, behavior of the animals, weight, orthopedic assessment and active lameness evaluation). The score of 10 results from our approach to use a summed score for the overall animal burden with emphasizing specific aspects differently. Since severe lameness would represent a major impact for the animal, score 10 was given.

However, we agree that it might be confusing within the present paper where only one part of our score is included. Furthermore, it did not influence the content of the paper since score 10 was never achieved. Therefore we changed the number and replaced 10 by 4 (also in Table 1 and the respective figures).

There is no presentation of the orthopedic score data in the result section of the manuscript. Either delete this section or include it in the result and compare with the data obtained with the pressure mat.

Thank you for this suggestion, we deleted the orthopaedic score.
